# 2176. *In Vitro* Evaluation of Delafloxacin Activity Against Contemporary US Isolates from Cystic Fibrosis Patients Hospitalized with Pneumonia: Results from the SENTRY Antimicrobial Surveillance Program (2019-2021)

**DOI:** 10.1093/ofid/ofac492.1796

**Published:** 2022-12-15

**Authors:** Dee Shortridge, Jennifer M Streit, Michael D Huband, Mariana Castanheira

**Affiliations:** JMI Laboratories, North Liberty, Iowa; JMI Laboratories, North Liberty, Iowa; JMI Laboratories, North Liberty, Iowa; JMI Laboratories, North Liberty, Iowa

## Abstract

**Background:**

Delafloxacin (DLX) is a broad-spectrum fluoroquinolone antibacterial approved in the US for the treatment of community-acquired bacterial pneumonia (CABP) and acute bacterial skin and skin structure infections. DLX is indicated to treat CABP caused by multiple pathogens, including methicillin-susceptible *Staphylococcus aureus* (MSSA) and *Pseudomonas aeruginosa* (PSA). *S. aureus* (SA) and PSA are common pathogens causing pneumonia in cystic fibrosis (CF) patients. In this study, the *in vitro* susceptibilities of DLX and comparator quinolones were determined for clinical isolates from US CF patients collected during 2019-2021.

**Figure:**
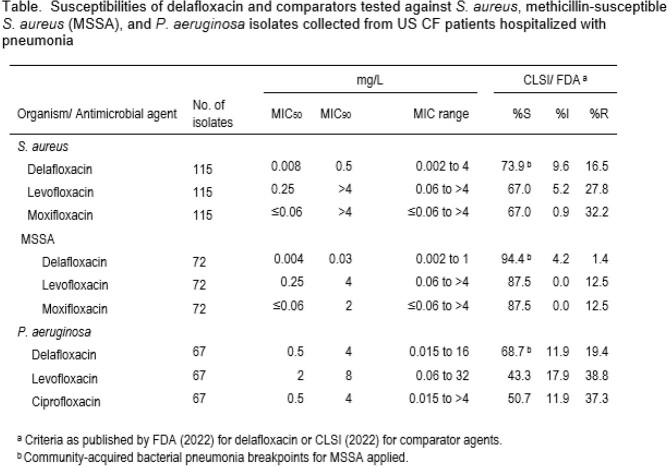

**Methods:**

Isolates from CF patients hospitalized with pneumonia were consecutively collected at 17 US medical centers participating in the SENTRY Surveillance Program. Sites submitted 1 isolate per patient per infection episode. Isolate identification was determined at each site and confirmed using MALDI-TOF at JMI Laboratories. Susceptibility testing was performed according to CLSI broth microdilution methodology. FDA interpretive criteria were used for DLX, and CLSI (2022) criteria were applied to comparators.

**Results:**

A total of 115 SA, including 72 MSSA and 67 PSA, were submitted. Susceptibilities (%S) to DLX, levofloxacin (LEV), and moxifloxacin (MOX) for MSSA are shown in the table. As MOX does not have breakpoints for PSA, ciprofloxacin (CIP) was tested. Against all SA, %S was 73.9%, 67.0%, and 67.0% for DLX, LEV, and MOX, respectively. DLX had the highest %S against MSSA (94.4%). The %S to LEV and MOX was 87.5% and 87.5%. DLX was also more active than comparators against PSA, with DLX 68.7%S, while LEV was 43.3%S and CIP was 50.7%S.

**Conclusion:**

DLX had good activity against recent CF isolates from US hospitals, and had the highest percent susceptibility of the quinolones tested against MSSA and PSA. These *in vitro* data suggest that DLX could be a useful therapy when coverage of both MSSA and PSA is needed.

**Disclosures:**

**Dee Shortridge, PhD**, AbbVie: Grant/Research Support|JMI Laboratory: Employee|Melinta: Grant/Research Support|Menarini: Grant/Research Support|Shionogi: Grant/Research Support **Jennifer M. Streit, BS, MT(ASCP)**, Cidara: Grant/Research Support|GSK: Grant/Research Support|Melinta: Grant/Research Support|Shionogi: Grant/Research Support **Michael D. Huband, BS**, AbbVie: Grant/Research Support|Melinta: Grant/Research Support **Mariana Castanheira, PhD**, AbbVie: Grant/Research Support|Cidara: Grant/Research Support|GSK: Grant/Research Support|Melinta: Grant/Research Support|Pfizer: Grant/Research Support|Shionogi: Grant/Research Support.

